# Prevention of donation-related infections: investigating the use of antibiotics in the decontamination of preservation fluid for organ transplantation

**DOI:** 10.3389/fcimb.2025.1572799

**Published:** 2025-07-23

**Authors:** Yazhe Duan, Junhao Yu, Pei Zhang, Yuhong Li, Kewen Chen, Yanfeng Li, Yuxiang Wan, Kang Wu, Li Zeng, Yanhua Li, Mingxing Sui

**Affiliations:** ^1^ Department of Organ Transplantation, The First Affiliated Hospital of Naval Medical University, Shanghai, China; ^2^ Department of Laboratory Diagnostics, Shanghai Changhai Hospital, Shanghai, China

**Keywords:** donation-related infection, CRGNB, decontamination, polymyxin, transplant

## Abstract

**Background:**

Donation-related infections (DRIs), particularly those caused by carbapenem-resistant gram-negative bacteria (CRGNB), can have disastrous consequences because of their extensive drug resistance. Contamination during graft acquisition and transport can lead to DRIs, and the use of antibiotics in preservation fluid (PF) before organ transplantation can reduce the incidence of DRIs. This study was to determine and compare the effectiveness of different PF decontamination regimens to prevent CRGNB related DRIs.

**Methods:**

Twelve CRGNB strains were chosen to be the targets of decontamination, and a drug concentration gradient was established for each test drug based on the previous clinical research. In addition the standard decontamination procedures were performed to evaluate the antimicrobial effectiveness of polymyxin B (PB), colistin sulfate (CS), colistimethate sodium (CMS) and amikacin (AK) in the 0~4°C PF, and to explore the antimicrobial effects of CMS after different preprocessing methods.

**Results:**

PB and CS exhibited significantly better antimicrobial effectiveness against CRGNB than AK and CMS in the 0~4°C PF, and the antimicrobial effects on CRGNB increased with the increasing concentration of drugs. Notably, CMS after pretreatment (CMS-AP), its antibacterial was significantly enhanced at 4°C.

**Conclusions:**

The PF decontamination is important in preventing the DRIs caused by CRGNB, and the decontamination regimens based on PB or CS were confirmed effective. Notably, CMS could even achieve a better decontamination effect than PB after a simple and fast pretreatment.

## 1 Introduction

Transplantation recipients are inevitably at risk for donation-related infections (DRIs) (Grąt et al., 2012; Yu et al., 2019; Corbel et al., 2020). Donation-related infections (DRIs) refer to infections that was possibly caused by pathogens from the donors or from the process of procurement, preservation, and transportation. Particularly, DRIs caused by carbapenem-resistant gram-negative bacteria (CRGNB) can cause disastrous consequences, including graft dysfunction, rejection, and even death. Preservation fluid (PF) contamination is defined as a positive result of preservation fluid culture. PF contamination are closely related to DRIs, and holding a great potential in the monitoring and intervention of the CRGNB transmission (Dębska-Ślizień et al., 2015; Li et al., 2022). Therefore, it is necessary to develop effective and economical decontamination programs (Jashari et al., 2007).

The application of antibiotic is crucial in decontamination protocols. In the previous study, a standardized decontamination protocol was designed and a clinical trial was conducted, which confirmed the decontamination protocol based on colistin sulfate (CS) was safe and could reduce the incidence of DRIs (Sui et al., 2022). However, the application of CS is greatly limited because it is not clinically accessible in most countries, while polymyxin B (PB) and colistimethate sodium (CMS) are more frequently utilized in clinical practice (El-Sayed Ahmed et al., 2020). At the same time, we interviewed large transplant centers in China. Amikacin (AK) is used as a routine decontamination protocol, so this study also used AK as a control study (Yu et al., 2019). PB and CS exhibit similar antimicrobial properties, and in certain instances PB has demonstrated slightly superior antimicrobial efficacy compared to that of CS (Evans et al., 1999; Cai et al., 2015; Doymaz and Karaaslan, 2019). CMS is an inactive prodrug without inherent antibacterial properties, and it can be converted quickly to produce antimicrobial effects when dissolved (Cai et al., 2015; Grégoire et al., 2017). Therefore, both PB and CMS also hold their potentials as PF decontamination antibiotics.

This study evaluated the antimicrobial effects of varying concentration gradients of PB, CMS, CS, and AK on CRGNB strains *in vitro*, to provide valuable references for a practical and efficient PF decontamination protocol.

## 2 Methods

### 2.1 Bacterial strains

Twelve CRGNB isolates were obtained from various clinical samples, including 3 strains of carbapenem-resistant Klebsiella pneumoniae (CRKP), 4 strains of carbapenem-resistant Pseudomonas aeruginosa (CRPA), 3 strains of carbapenem-resistant Acinetobacter baumannii (CRAB), and 2 strains of carbapenem-resistant Escherichia coli (CREC). These strains were collected from inpatients in eight different departments of Shanghai Changhai Hospital, including the emergency room, intensive care unit, urology ward, cerebrovascular disease center, hematology ward, vascular surgery ward, burn unit, and organ transplant ward. The specimens included blood, sputum, urine, cerebrospinal fluid, and wound secretions from various anatomical sites. All bacterial strains were isolated and cultured in the microbiology laboratory following the National Clinical Laboratory Practice Guidelines (4th edition). The bacteria were identified using the Bruker Daltonik MALDI Biotyper system (Germany). Drug susceptibility test was performed using the paper disc diffusion method, drug susceptibility test equipment: Vitek 2 system (bioMerieux, France). All experiments were performed with three iterations of validation.

### 2.2 Genetic testing for drug resistance

After the bacteria were isolated and cultured from specimens, individual bacterial colonies were inoculated into the designated broth media. After growth verification, genetic testing was performed on all samples via fluorescent PCR. This procedure was to authenticate the genotype and phenotype of the bacterial strains and detect the presence of antibiotic resistance genes.

### 2.3 Antimicrobial susceptibility testing

The microbroth dilution method was used to determine the minimum bactericidal concentration (MBC) of each drug against bacterial strains at 0-4°C, and the result is referred to as the 4°C-MBC (Nakaminami et al., 2019).

Before the experiment, the strain was recovered on blood agar media and passage culture was performed. After 18–24 hours of passage culture, we selected single colonies for inoculation in PF. Each bacterial suspension was prepared at a concentration of 1.5×10^^8^ cfu/ml using a McClure turbidimeter. The liquid medium was prepared using CA-MH broth medium powder, and the pH was maintained at 7.3 ± 0.1. After autoclaving, solutions containing 512 µg/ml PB, CS, CMS, or AK were prepared using the same liquid media (for PB and CS, the following conversion factors were used: PB: 1 mg = 10,000 U; and CS: 1 mg = 30,000 U). Afterward, the four antibiotic solutions were diluted using the multiplicative dilution method to generate 11 solutions with concentrations ranging from 0.5 to 512 µg/ml.

Then, 100 µl of each dilution of antibiotic solution and 10 µl of bacterial suspension were added to a 96-well plate, and negative control wells (without bacteria) and positive control wells (without antibiotics) were set up as well. The prepared 96-well plate was incubated at low temperature (0-4°C) for 24 hours. After incubation, 1 μl of liquid was aspirated from each well and transferred to a new 96-well plate. The new plate was washed with 100 μl of sterile deionized water to remove any antibiotics that may have been carried over. Then, CA-MH broth medium without antibiotics was added to another 96-well plate. One microliter of the rinsed liquid was removed, transferred to the CA-MH broth medium without antibiotics. After thorough mixing, this plate was incubated at 37°C for 24 hours. The lowest concentration of the antimicrobial drug that resulted in no observable colony growth in the 96-well plate was designated the 4°C-MBC of the drug against the bacteria.

### 2.4 *In Vitro* decontamination test

The same steps as mentioned above were followed to prepare a bacterial suspension at a concentration of 3×10^^4^ cfu/ml. In the previous retrospective study, a simple regimen of dissolving CS (500,000 U) in 1000 ml (2× 500 ml) of HC-A II solution (hypertonic citrate adenine solution II, Shanghai Haini Pharmaceutical Co., Ltd., Shanghai, China) was utilized in transplant recipients, and this regimen could reduce the incidence of PF-associated CRGNB-DRIs (Sui et al., 2022). Based on this study, it is defined that the doses of CS (500,000 U Shanghai SPHNewAsiaPharmaceuticalCo., Ltd., Shanghai, China), PB (500,000 U Shanghai Shangyao First Biochemical Pharmaceutical Co., Ltd.,Shanghai, China), CMS (150 mg Nanjing Chia-Tai Tianqing Pharmaceutical Company), and AK (120 mg Zhejiang Taikang Pharmaceutical Group Co., Ltd.,Zhejiang, China) dissolved in 1,000 ml of PF as the base concentration, and then dispensed 4-fold, 2-fold, 1-fold, and 1/2-fold dilutions. Then, the bacterial suspension and antibiotic solution were mixed in a 96-well plate. Different concentrations of the antibiotic solution were added from high to low concentration (50 μl per well), and then 50 μl of bacterial suspension was added to each well, followed by mixing to homogeneity. The final concentration of the bacterial solution was set to 1.5×10^^4^ cfu/ml, and the final concentration of each drug was 4-fold, 2-fold, 1-fold, or 1/2-fold the base concentration.

The spiked 96-well plates were incubated in a refrigerator at 0-4°C for 2–4 hours. Then, a 10 µl sample was taken from each well for inoculation into blood agar plates. The plates were incubated at 37°C in a constant temperature box for 18–20 hours, after which the growth of the colonies on the media was observed, and the colonies were counted. The experiment was repeated three times.

### 2.5 CMS preprocessing

The *in vitro* hydrolysis of CMS is influenced by various factors, with temperature being the main factor (Wallace et al., 2008; Dudhani et al., 2010; Imberti et al., 2012). Several preprocessing regimens, including different hydrolysis temperatures and different time, were experimented to observe the inhibitory efficiency of CMS at the base concentration. After considering the efficiency and feasibility, the prepared CMS solutions would be heated in a water bath at 60°C for 15 min or 30 min, and then were cooled to room temperature (25°C) before the *in vitro* decontamination simulation experiment.

### 2.6 Statistical analysis

SPSS 25.0 software was used for data analysis and GraphPad Prism 9 software was used for data analysis and figures processing. The drug main effect and concentration main effect were statistically inferred by factorial design multivariate analysis of variance. Multivariate analysis of variance (ANOVA) with factorial design was used for comparison between antibiotic groups, and univariate ANOVA or Kruskal-Wallis H test was used for comparison within antibiotic groups according to the type of data distribution. To minimize the impact of slight differences in bacterial counts between the blank control and experimental groups in different experimental sets, we expressed the antimicrobial effect of each drug as an antimicrobial rate. The antimicrobial rate was calculated based on the number of bacterial colonies as follows: antimicrobial rate *1 = (A - B)/A × 100%, where A represents the bacterial colony count in the positive control group and B represents the bacterial colony count in the experimental group.

## 3 Results

### 3.1 Genetic testing

The genetic testing data showed that the main resistance genes in the strains tested in this study were OXA-23 for CRAB, NDM and CTX-M for CREC, and KPC, NDM and CTX-M for CRKP. See **Table 1** for details.

**Table 1:** Genetic testing for drug resistance.

Resistance gene Strains	Resistance gene 1	Resistance gene 2	Resistance gene 3
22092811			
22092510			
22092805			
22092719			
CRAB			
22092919	OXA-23		
22092509	OXA-23		
22092506	OXA-23		
CREC			
22092808	NDM		
22101402	CTX-M		
CRKP			
22092608	CTX-M	KPC	
22092215	CTX-M	KPC	
2.02E+08	CTX-M	KPC	NDM

### 3.2 Antimicrobial effects of different antibiotics on CRGNB

The CRKP, CRAB, CRPA and CREA strains in HC-A II PF were decontaminated *in vitro* at 0-4°C using different concentrations of four antibiotics before colony counting and subsequent calculation of the antimicrobial rate.

**Figure 1:**
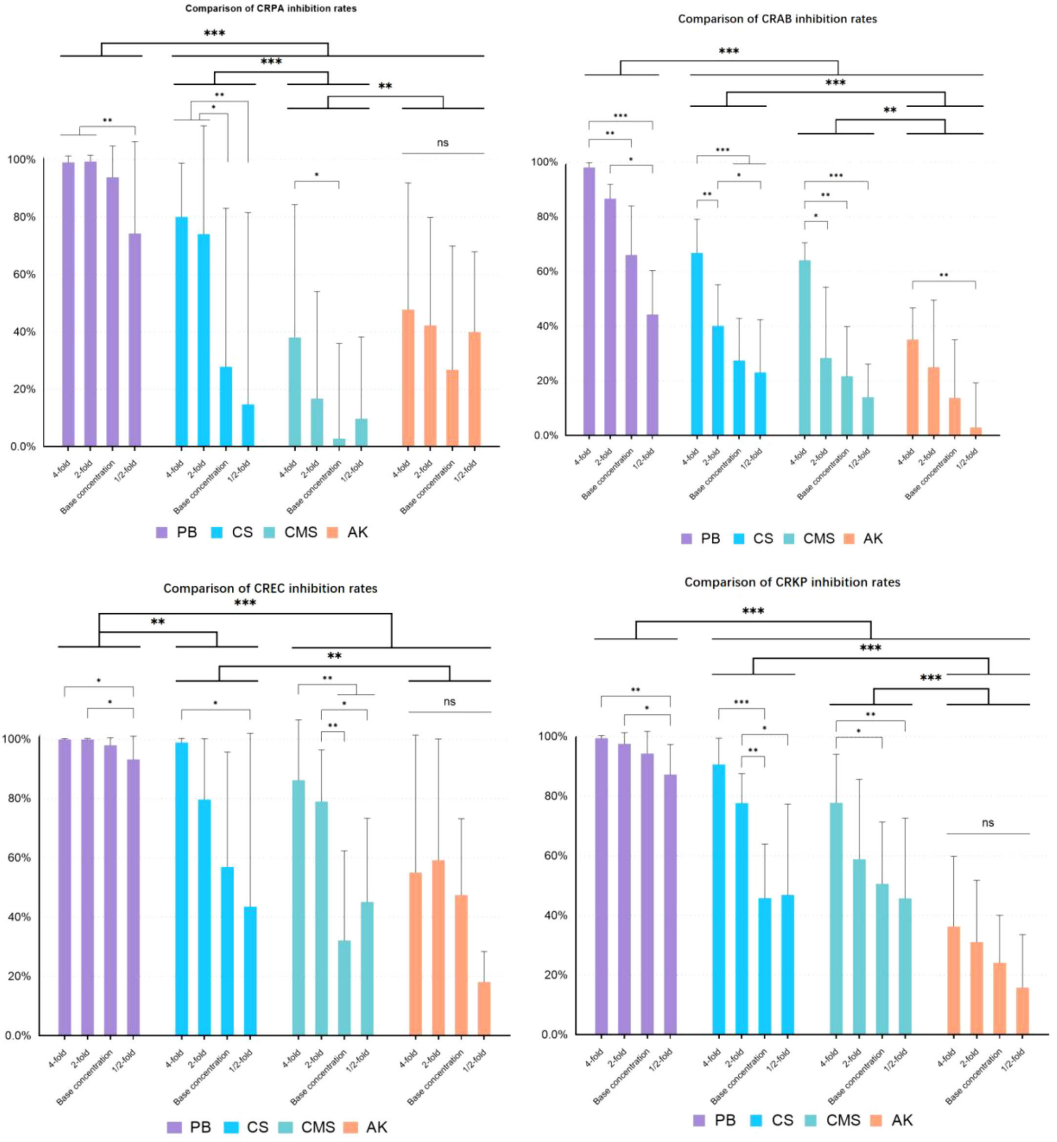
Antimicrobial effects of different antibiotics on CRGNB. CRGNB: Carbapenem-Resistant Gram-Negative Bacteria. The bacterial concentration was 1.5*10^^4^ cfu/ml. The drug concentration gradient of PB was 4000U/ml, 2000U/ml, 1000U/ml, 500U/ml. The drug concentration gradient of CS was 4000U/ml, 2000U/ml, 1000U/ml, 500U/ml. The drug concentration gradient of CMS was 1200μg/ml, 600μg/ml, 300μg/ml, 150μg/ml. The drug concentration gradient of AK was 1600μg/ml, 800μg/ml, 400μg/ml, 200μg/ml. * p<0.05, ** p<0.01, *** p<0.001.

In each of the four CRGNB groups, the drug presented a significant main effect (*p*<0.001), as did the concentration (*p*<0.005), and no significant interaction effect was observed between the drug and concentration (*p*>0.05) (As shown in **Figure 1**).This indicates that different antibiotics do not show great differences in inhibition rate due to changes in concentration. PB achieved the strongest inhibitory effect on all the obtained CRGNB strains, CS was relatively less effective, and CMS and AK presented poor antimicrobial effect. Generally, the antimicrobial effect increased with increasing drug concentration, with the greatest effect observed at the 4-fold concentration and the least effect observed at the 1/2-fold concentration.

### 3.3 The 4°C-MBC values for each antibiotic

The 4°C-MBC values of each antibiotic against the different strains of bacteria are shown in **Table 2**, and these data are essentially consistent with the antimicrobial rates of the corresponding antibiotics. PB was found with the lowest 4°C MBC, CS also showed a greater advance than CMS and AK in all strains. All the four antibiotics, were relatively weak against CRAB, and the 4°C MBC of PB was only 64μg/ml.

**Table 2:** 4°C minimum bactericidal concentration (MBC).

Antibiotics Strains	AK	CMS	PB	CS
CRPA				
22092811	512	>512	4	4
22092510	>512	>512	8	16
22092805	>512	>512	4	8
22092719	>512	256	4	8
CRAB				
22092919	>512	>512	128	256
22092509	>512	256	64	256
22092506	>512	512	64	512
CREC				
22092808	>512	256	16	64
22101402	>512	128	8	4
CRKP				
22092608	>512	>512	128	256
22092215	>512	>512	64	256
2.02E+08	>512	>512	4	8

MBC: Minimum Bactericidal Concentration. PB: polymyxin B, CS: colistin sulfate, CMS: colistimethate sodium, AK: amikacin. CRKP: Carbapenem-Resistant Klebsiella Pneumoniae, CRPA: Carbapenem-Resistant Pseudomonas Aeruginosa, CRAB: Carbapenem-Resistant Acinetobacter Baumannii, CREC: Carbapenem-Resistant Escherichia Coli. The bacterial concentration was 1.5*10^4 cfu/ml. The drug concentration gradient was 512μg/ml, 256μg/ml, 128μg/ml, 64μg/ml, 32μg/ml, 16μg/ml, 8μg/ml, 4μg/ml, 2μg/ml, 1μg/ml, 0.5μg/ml. (for PB and CS, use the conversion factor PB: 1mg = 10,000 U; CS: 1mg = 30,000 U).

### 3.4 Antimicrobial effects of CMS after pretreatment

**Figure 2:**
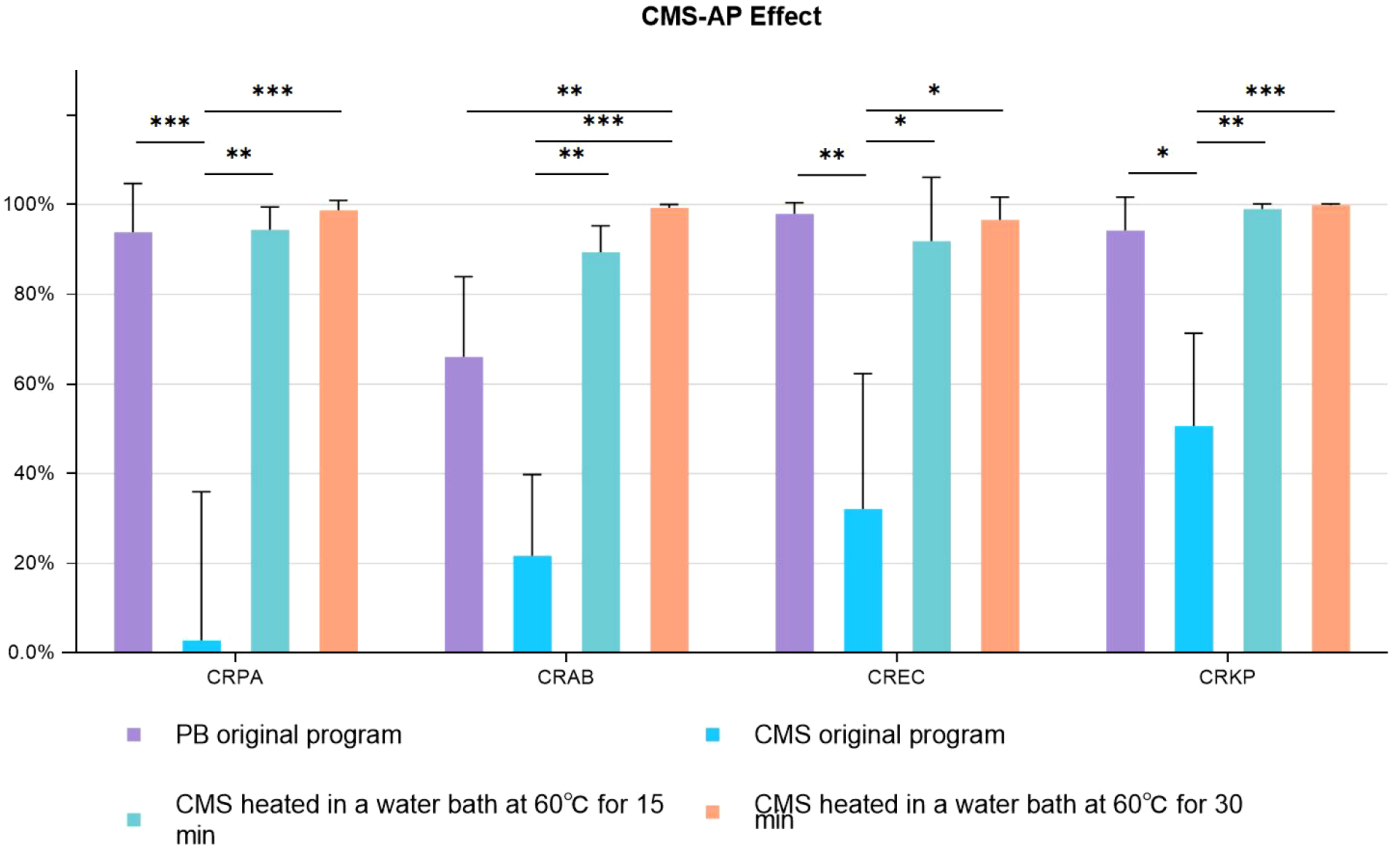
Antimicrobial effects CMS-AP CMS-AP: Colistimethate sodium After Pretreatment. The bacterial concentration was 1.5*10^^4^ cfu/ml. The drug concentration gradient of CMS was 300ug/ml (Base concentration). The drug concentration gradient of PB was 1000U/ml (Base concentration); * p<0.05, ** p<0.01, *** p<0.001..

CMS was heated in a 60°C water bath for 15 minutes or 30 minutes before the simulated decontamination process *in vitro*. The result is shown in **Figure 2**. It was observed that the CMS without pretreatment was ineffective on CRGNB in the PF decontamination, with a poor antibacterial effect at only 2.81% (93.86% with PB) on CRPA and at 50.61% (94.28% with PB) on CRKP. The antibacterial effect of CMS-AP at basic concentration on CRGNB was vastly improved, and there was no significant difference between the two pretreatment methods (15 minutes VS 30 minutes). The antibacterial rate of CMS-AP was even better than PB in every group, and particularly much significant in the group of CRAB (66.05% VS 89.33%, *p*<0.01).

## 4 Discussion

PF contamination is prevalent during organ preservation and transportation, with varied incidence rate from 2.2% to 92% (Veroux et al., 2010; Grąt et al., 2012; Oriol et al., 2016). Alessandra Mularoni (Mularoni et al., 2024) reported that the incidence of CR-GNB-related infection after organ transplantation reached 1.4%. Therefore, decontaminating the graft PF is considered critical during organ transplantation, as it has a direct impact on decreasing the rate of DRIs (Picola Brau et al., 2024), but no standardized PF decontamination regimen has been currently well received. Some centers preferred the broad-spectrum antibiotics, but most of them were not targeted to the CRGNBs, which were the leading cause of severe DRIs. This study focused on the DRI caused by four devastating CRGNB, and OXA-23, KPC and NDM were found to be the main drug resistance genes (Iovleva and Doi, 2017; Bose et al., 2022). These resistance genes mainly produce the corresponding β-lactamase, which enables bacteria to hydrolyze β-lactam antibiotics and thus develop resistance to these antibiotics (De Angelis et al., 2020). The results suggested that the broad-spectrum antibiotic AK performed the poorest antimicrobial effect in the low temperature PF, indicating that AK did not target at these CRGNB, should not be included in the decontamination regimen.

Due to the lack of effectively and economically beneficial antibiotics, polymyxin antibiotics have been reintroduced into the clinical practice and considered as one of the last resorts, especially in the treatment of CRGNB (Kagami et al., 2021; Ahumada Topete et al., 2023). In this study, PB presented with greater than 90% antimicrobial effect of CRPA, CRKP, and CREC and with only 66.05% of CRAB at the base concentration, which may be related to the production of the Ambler D-like serine hydrolase OXA-23 by CRAB (Barin et al., 2013). CS showed the second strongest antimicrobial effect in all the selected CRGNB strains at different concentrations, and it should be noted that only the high concentration (4-fold) of CS could achieve relatively satisfactory results. Similar to PB, CS also was inadequate against CRAB, even at the 4-fold concentration.

Compared with PB and CS, CMS is widely utilized with plenty of clinical evidence. CMS is reported to be effective against CRGNB after intravenous administration (Wang et al., 2020), as well as in the other routes of administration, such as atomized inhalation, because CMS could rapidly convert to CS upon dissolution (Dijkmans et al., 2014; Zhu et al., 2021). However, this *in vitro* study showed that the bactericidal effect of CMS was well below expectations in the low temperature PF. Research conducted by Dudhani RV et al. suggested that the *in vitro* antibacterial effect of CMS is associated with the solution’s temperature, concentration, and solvent composition (Wallace et al., 2008; Dudhani et al., 2010; Imberti et al., 2012). Therefore, combinations of different solvent temperatures, different solute concentrations and different pre-heating time were included in this study to further improve the *in vitro* hydrolytic conversion efficiency of CMS. It was observed that the conversion might be greatly influenced by the low-temperature environment. After conducting a series of experiments, it was discovered that a 60°C water bath of CMS (at basal concentration) for either 15 or 30 minutes greatly enhanced its bactericidal properties. Interestingly, these two treatment methods did not present a significant impact on the antibacterial effectiveness, indicating a 15 minutes of pretreatment could achieve a satisfactory activation of CMS and CMS-AP could be a relative simple and feasible option for PF decontamination. The antibacterial rates of CMS-AP against CRGNB exceeded 90%, a substantial improvement compared to the poor results of untreated CMS. The antibacterial rates of CMS-AP against CRPA, CRKP, and CRAB were found to be even slightly higher than those of PB. Notably, the effect of PB in CRAB was less satisfactory (66.05%), and the inhibition effect against CRAB of CMS-AP was notably higher than that of PB (*p*<0.01), providing compelling evidence for the use of CMS-AP for the PF decontamination.

## 5 Limitations

This experiment was conducted under *in vitro* conditions using bacterial cultures and antibiotics to simulate a clinical decontamination environment. However, practical clinical decontamination procedures usually involve additional steps such as irrigation. Therefore, the experimental results do not completely accurately represent the efficacy of these antibiotics during clinical decontamination. Furthermore, in actual clinical practice, infections caused by multidrug-resistant bacteria normally necessitate combination therapy with multiple antibiotics to enhance efficacy and decrease the likelihood of resistance. In this study, we only assessed the effects of a single drug. Therefore, it is necessary to further explore the combination treatment options to improve the antimicrobial effect in the decontamination regimen.In this study, we focused on four of the most representative CRGNB strains as the test strains. In clinical Settings, however, a wide variety of bacterial species exist, and reports of fungal and virus-caused DRIs are not uncommon. Therefore, further research with a larger sample size is needed. In addition, the number of strains expressing NDM enzymes was too small, and further expansion of the sample size is needed to verify the effectiveness of the polymyxin antibiotics.

Several reports have shown that nephrotoxicity and neurotoxicity can result after the intravenous injection and inhalation of Polymyxin antibiotics,in the clinic (Wagenlehner et al., 2021). In this study, we aimed to explore the antimicrobial effects of Polymyxin antibiotics,*in vitro* at low temperature, but nephrotoxicity under these conditions was not investigated. In fact, in our previous clinical study (Sui et al., 2022), no obvious nephrotoxic or neurotoxic effects of polymyxin antibiotics were observed after the decontamination *in vitro*, which to some extent could indicate the safety of decontaminating PF with CS. However, further decontamination experiments involving animal models are essential to confirm the safety of polymyxin antibiotics.

## 6 Conclusions

The goal of decontamination should be targeted to decrease the risk of CRGNB related DRIs. The CMS-AP and PB presented satisfactory antimicrobial effects against CRGNB, holding great potential to be the effective and feasible decontamination option in clinical practice.

## Data Availability

The original contributions presented in the study are included in the article/supplementary material. Further inquiries can be directed to the corresponding authors.
